# KUFfed by drought: A KARRIKIN-upregulated F-box protein compromises plant growth and survival under drought

**DOI:** 10.1093/plphys/kiac413

**Published:** 2022-09-05

**Authors:** Marieke Dubois

**Affiliations:** Department of Plant Biotechnology and Bioinformatics, Ghent University, Ghent, Belgium; VIB Center for Plant Systems Biology, Ghent, Belgium

During their life cycle, plants experience a wide variety of environmental stresses that could compromise their growth and development or even threaten their survival. Plants have evolved sophisticated mechanisms to respond and, ultimately, adapt their morphology to such adverse environmental conditions. Drought, for example, is a complex abiotic stress causing worldwide agricultural yield losses every year (Farooq et al., 2009). When plants perceive moderate drought stress, one of the first morphological adaptations is to reduce shoot growth and sustain root growth to increase water uptake by the roots while limiting water evaporation from the shoot (Verslues, 2006). Plants also close stomata and increase the biosynthesis of a wax layer to protect the leaves. In case drought stress becomes more severe, protective solutes are synthesized to limit intracellular damage (Verslues, 2006).

During the last decades, the vast majority of drought stress research aimed to increase the plant’s resilience to severe drought stress by boosting the protective mechanisms and the plant’s sensitivity to abscisic acid (ABA), a central phytohormone in drought resistance. While many such “over-protected” lines are resistant to severe drought, they show stunted growth and reduced yield under normal conditions or under moderate level of drought (Skirycz et al., 2011). Plant growth and defense to drought stress are both energy-demanding processes, explaining why there is a growth tradeoff when stress defense is artificially boosted (Claeys and Inzé, 2013). Currently, improving plant growth and yield under moderate drought stress is considered a better engineering goal than achieving defense to more severe drought (Simmons et al., 2021; Tenorio Berrío et al., 2022). Nevertheless, the number of lines with this beneficial phenotypic trait is still very limited, and the molecular players that could potentially affect both growth under moderate drought and survival to extreme drought currently remain unexplored.

In the current issue of *Plant Physiology*, Tian et al. (2022) characterized the role of KARRIKIN-UPREGULATED F-BOX 1 (KUF1) in plant resistance to moderate and extreme drought. KUF1 is an F-box protein that is induced in Arabidopsis (*Arabidopsis thaliana*) plants treated with karrikin, a bioactive molecule derived from burning vegetation (Nelson et al., 2010). Although the exact molecular mechanism is still unclear, karrikin initiates a signaling cascade involving a receptor, KARRIKIN-INSENSITIVE 2 (KAI2), and the F-box protein MORE AXILLARY GROWTH 2 (MAX2), which also contributes to the response to strigolactones, a phytohormone with a partially similar structure as karrikin (for a review, see Wang et al., 2022). Interestingly, KAI2 has previously been linked to plant growth and drought tolerance in Arabidopsis (Li et al., 2017). Mutants of *kai2* were less sensitive to karrikin and showed increased biomass under normal conditions, coupled with reduced growth under moderate drought and increased lethality under extreme drought stress (Figure 1).

**Figure 1:**
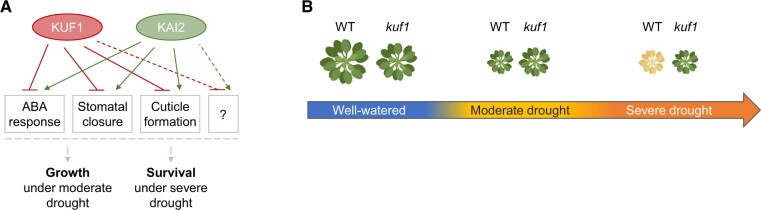
A schematic representation of the role of KUF1 in response to environmental stresses. A, Tian et al. (2022) identified KUF1 as a negative regulator of several physiological processes associated with drought resistance, including stomatal closure, ABA responsiveness, cuticle formation, and possibly other downstream targets that remain unidentified. KAI2, a receptor in the karrikin pathway, was previously found to have functions opposite to KUF1 (Li et al., 2017). B, In well-watered conditions, Arabidopsis seedlings in which the *KUF1* gene was knocked out show slightly reduced biomass compared to wild-type (WT) plants (Tian et al., 2022). Under moderate drought, *kuf1* mutants showed less biomass reduction than WT plants. Upon severe drought stress, *kuf1* mutants survived on average four-fold better than WT plants. Made with Biorender.com.

Here, Tian et al. (2022) profiled the *kuf1* mutant, which shows constitutive karrikin responses (Sepulveda et al., 2022). Compared to wild-type plants, the *kuf1* mutant displayed over four-fold increase in survival rates upon exposure to severe drought stress (Tian et al., 2022; Figure 1). In addition, the *kuf1* mutant was also more tolerant to moderate drought, showing less biomass (dry weight) decrease under drought (Figure 1). Physiologically, the better performance of the *kuf1* mutant under the two levels of drought severity could be attributed to a reduced stomatal aperture in the leaves and, thus, slower leaf water loss upon drought. Reduced stomatal opening and water loss could, in turn, result from the hypersensitive ABA response that was observed in the *kuf1* mutant. To get more insights in additional mechanisms that could explain the improved resistance of the *kuf1* mutant, the authors profiled the *kuf1* transcriptome under well-watered conditions and dehydration stress. Genes related to the biosynthesis of lipids and fatty acids were enriched in the *kuf1* mutant, suggesting that the *kuf1* mutant contains more cuticular wax. As predicted by the transcriptome data, the authors found more epicuticular wax crystals on young stems of *kuf1* compared to the wild type, a difference that was even more pronounced under drought stress. Overall, the molecular and physiological analyses performed in this study demonstrated that KUF1 has a negative effect on plant growth and survival under moderate and severe drought stress conditions, respectively (Figure 1).

Taken together, the findings of Tian et al. (2022) pave a way toward engineering plants that are more resistant to different severities of drought. Many mutants identified in the past were resistant to severe drought but did not perform better than wild-type plants in terms of biomass production under mild drought (Skirycz et al., 2011; Tenorio Berrío et al., 2022). The *kuf1* mutant shows slightly reduced biomass under well-watered conditions, which, from an agricultural point of view, makes the engineering of KUF1 less attractive despite the drought-resistant phenotype of the mutant. However, one way to circumvent the dwarfism caused by knocking out *KUF1* could be to knockdown *KUF1* in a drought-induced manner. This would allow normal plant growth under well-watered conditions while maintaining the beneficial traits resulting from KUF1 inactivation under drought stress conditions. Alternatively, boosting drought resistance while avoiding the stunted growth under normal conditions might be achievable by engineering specific downstream targets of KUF1. Interestingly, the KUF1 protein is part of the signaling pathway of karrikins, of which many aspects are still unexplored. It is possible that karrikin responses, or specific proteins acting as downstream targets of the KUF1 F-box protein, play a role in maintaining plant growth under moderate drought and in protecting the plant against more severe drought. Identifying these downstream targets and investigating their specific role in drought stress tolerance will be an exciting direction for future research.

## Funding

M.D. is a postdoctoral fellow of Flanders Research Foundation (FWO-12Q7919N).

*Conflict of interest statement*. None declared.
